# Identified novel heterozygous *HTRA1* pathogenic variants in Chinese patients with *HTRA1*-associated dominant cerebral small vessel disease

**DOI:** 10.3389/fgene.2022.909131

**Published:** 2022-08-10

**Authors:** Mei-Jiao Chen, Yi Zhang, Wen-Jiao Luo, Hai-Lin Dong, Qiao Wei, Juan Zhang, Qi-Qi Ruan, Wang Ni, Hong-Fu Li

**Affiliations:** ^1^ Department of Neurology and Research Center of Neurology in Second Affiliated Hospital, Zhejiang University School of Medicine, Key Laboratory of Medical Neurobiology of Zhejiang Province, Hangzhou, China; ^2^ Department of Neurology, The First Affiliated Hospital of Zhejiang Chinese Medical University, Hangzhou, China; ^3^ Department of Neurology, Shangyu People’s Hospital, Shaoxing, China

**Keywords:** cerebral small vessel disease, HTRA1, heterozygous variant, autosomal dominant, CARASIL

## Abstract

**Background:** Homozygous and compound heterozygous mutations in *HTRA1* cause cerebral autosomal recessive arteriopathy with subcortical infarcts and leukoencephalopathy (CARASIL). Recently, heterozygous pathogenic variants in *HTRA1* were described in patients with autosomal dominant cerebral small vessel disease (CSVD). Here, we investigated the genetic variants in a cohort of Chinese patients with CSVD.

**Methods:** A total of 95 Chinese index patients with typical characteristics of CSVD were collected. Whole exome sequencing was performed in the probands, followed by Sanger sequencing. Pathogenicity prediction software was applied to evaluate the pathogenicity of the identified variants.

**Results:** We detected five heterozygous *HTRA1* pathogenic variants in five index patients. These pathogenic variants included four known variants (c.543delT, c.854C>T, c.889G>A, and c.824C>T) and one novel variant (c.472 + 1G>A). Among them, c.854C>T, c.824C>T, and c.472 + 1G>A have never been reported in China and c.889G>A was once reported in homozygous but never in heterozygous. Three of them were distributed in exon 4, one in exon 2, and another splicing variant in intron 1. Four out of five probands presented typical features of CARASIL but less severe. The common clinical features included lacunar infarction, cognitive decline, alopecia, and spondylosis. All of them showed leukoencephalopathy, and the main involved cerebral area include periventricular and frontal area, centrum semiovale, thalamus, and corpus callosum. Anterior temporal lobes and external capsule involvement were also observed. Three probands had intracranial microbleeds.

**Conclusion:** Our study expanded the mutation spectrum of *HTRA1*, especially in Chinese populations, and provided further evidence for “hot regions” in exon 1–4, especially in exon 4, in heterozygous *HTRA1* pathogenic variants*.* Our work further supported that patients with heterozygous *HTRA1* pathogenic variants presented with similar but less-severe features than CARASIL but in an autosomal dominantly inherited pattern.

## 1 Introduction

Cerebral small vessel diseases (CSVDs) are a series of diseases that affect the small arteries, arterioles, venules, and cerebral capillaries. The main manifestations of CVSD include stroke and cognitive impairment (Verdura et al., 2015; Thaler et al., 2018; Wu et al., 2018; Zhang et al., 2018). Typical neuroimaging features include lacunar infarcts, white matter lesions, cerebral microbleeds, prominent perivascular spaces, and atrophy (Verdura et al., 2015; Pati and Battisti, 2018). Although most CSVD cases are sporadic and related to vascular risk factors, a minority of patients were identified with monogenic mutations (Verdura et al., 2015; Di Donato et al., 2017). Autosomal and X-linked inheritance patterns in either a recessive or dominant manner have been illustrated (Verdura et al., 2015; Pati and Battisti, 2018). Cerebral autosomal dominant arteriopathy with subcortical infarcts and leukoencephalopathy (CADASIL), which is caused by *NOTCH3* mutations, is the most common hereditary CSVD (Choi et al., 2005; Tikka et al., 2014; Wang, 2018).

Homozygous or compound heterozygous mutations of *HTRA1* gene are considered to cause cerebral autosomal recessive arteriopathy with subcortical infarcts and leukoencephalopathy (CARASIL) (Verdura et al., 2015; Wu et al., 2018). CARASIL is characterized by recurrent strokes and early onset cognitive decline with rapid progression. It is usually accompanied with alopecia, lumbago, or spondylosis deformans (Nozaki et al., 2015; Roeben et al., 2016; Ito et al., 2018; Favaretto et al., 2019). Multiple lacunars and white matter lesions are common in brain magnetic resonance imaging (MRI) of CARASIL patients. Pathologic features of CARASIL are fibrosis of the adventitia and loss of smooth muscle cells (SMC), multilayering and splitting of the elastic lamina, intimal thickening in cerebral small vessels (Ito et al., 2018). Nevertheless, recent studies showed that heterozygous *HTRA1* mutations could manifest as familial CSVD in autosomal dominant pattern, accounting for roughly 5% of hereditary CSVD in European population (Lee et al., 2018). The clinical features differ from typical CARASIL by the absence of extra-neurological symptoms, such as alopecia and lumbago, and a later age of onset (Verdura et al., 2015).

*HTRA1* is a repressor of TGF-β signaling which results in reduced amounts of mature TGF-β in the extracellular matrix (ECM) through cleaving proTGF-β1 in the endoplasmic reticulum. *HTRA1* mutations were demonstrated to result in greater amounts of mature TGF-β and upregulation of TGF-β signaling. TGF-β plays a key role in vascular remodeling via upregulating the production of fibroblast growth factor and connective tissue growth factor (Tikka et al., 2014).

Human *HTRA1* consists of nine exons and encodes a protein comprising four functional domains including an insulin-like growth factor binding protein domain, a Kazal-type serine protease inhibitor domain, a trypsin-like serine protease domain, and a PDZ domain (Tikka et al., 2014; Kang and Kim, 2015; Wu et al., 2018). The exons 3–6 encode the trypsin-like serine protease domain which inhibits signaling through TGF-β family members (Tikka et al., 2014). In 2009, Hara et al. indicated a link between inhibition of signaling by the TGF-β family members and ischemic CSVD, spondylosis, and alopecia (Hara et al., 2009). Lee et al. further confirmed that the impaired *HTRA1* protease activity seems to be at the center of *HTRA1*-related autosomal dominant CSVD (Lee et al., 2018). However, the role of deleterious *HTRA1* pathogenic variants is not fully understood yet.

Here, we reported five CSVD pedigrees with heterozygous *HTRA1* mutations and summarized their clinical features.

## 2 Materials and methods

### 2.1 Study subjects

#### 2.1.1 Ethics statement

This study was approved by the Ethics Committees of Second Affiliated Hospital of Zhejiang University School of Medicine. Written informed consents were obtained from all the participants or their legal guardians.

#### 2.1.2 Included subjects

A consecutive series of 95 unrelated cases with typical characteristics of CSVD were enrolled from the Department of Neurology between March 2015 and July 2021. Clinical and neuroimaging data were retrospectively analyzed. The clinical features and neuroimaging were evaluated by at least two senior neurologists. The inclusion criteria were as follows: 1) aged between 18 and 70, 2) marked leukoaraiosis (grade 2–3 of the Fazekas scale) in neuroimaging (Fazekas et al., 2002), and 3) at least one of the following characteristics: lacunar infarction or transient ischemic attack, cognitive decline, or a family history of ischemic stroke or vascular dementia. In addition, 200 individuals of Chinese ancestry with no history of neurological symptoms were enrolled as matched controls.

### 2.2 Whole-exome sequencing, bioinformatic analysis, and Sanger sequencing

Genomic DNA from 95 patients was extracted from peripheral EDTA-treated blood using a QIAamp DNA Blood Minikit (QIAGEN, Hilden, Germany). Then the Agilent SureSelectTM Human All Exome V6 kit (Agilent Technologies Inc, Canada) on an Illumina Hiseq X Ten Analyzer (Illumina, United States) was used to perform WES in all the subjects. Frequency of all variants in general population was obtained from gnomAD, 1000 Genomes Project, and ExAC. *In silico* software, SIFT, PolyPhen-2, Mutation Taster, and CADD were used to predict the pathogenicity of identified variants. Variants were finally classified according to American College of Medical Genetics and Genomics (ACMG) guidelines. Sanger sequencing was performed to validate the variants analyzed by WES in patients and their available familial members. Co-segregation analysis was conducted in the pedigrees. The identified variants were also screened in age- and sex-matched control subjects.

### 2.3 Neuroimaging techniques

Neuroimaging features of patients carrying *HTRA1* mutations were analyzed through brain MRI, magnetic resonance angiography (MRA), or computed tomography angiography (CTA) of intra-extracranial vessels.

## 3 Results

### 3.1 Identification of pathogenic variants by WES and Sanger sequencing

**FIGURE 1:**
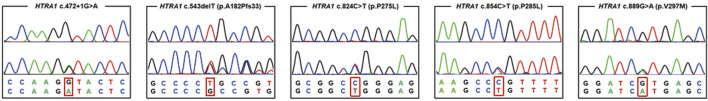
Chromatograms of five pathogenic variants identified in the present study. The upper chromatogram in each frame represents the reference sequence, and the lower one depicts the mutant sequence (c.472 + 1 G > A, c.824C>T, c.854C>T, c.543delT, c.889G>A in *HTRA1* gene).

After performing WES in the 95 probands with CVSD, we excluded pathogenic variants of other CSVD causative genes, including 39 pathogenic variants in *NOTCH3*, 3 pathogenic variants (c.1379A>G, c.3431C>G, c.4560G>A) in *COL4A1*, 1 pathogenic variant (c.3586C>T) in *COL4A2*, and 1 pathogenic variant (c.1013G>A) in *TREX1*. We finally detected 5 heterozygous *HTRA1* pathogenic variants in 5 probands after verification by Sanger sequencing. Four variants were previously reported including c.543delT (p.A182Pfs*33), c.824C>T (p.P275L), c.854C>T (p.P285L), c.889G>A (p.V297M) while c.472 + 1G>A is a novel variant (Figure 1). Among them, c.854C>T, c.824C>T, and c.472 + 1G>A have never been reported in China and c.889G>A was once reported in homozygous condition but never in heterozygous. In addition, the variants c.543delT, c.824C>T, c.854C>T, and c.889G>A reside in an evolutionarily conserved region of *HTRA1* (Supplementary Figure S1).

The splicing variant c.472 + 1G>A was absent in ExAC, 1000G, genomAD, and 200 matched controls. It was predicted to be deleterious by Mutation Taster. Human Splicing Finder reveals that this variant causes the alteration of the WT donor site and most probably affects splicing. According to ACMG, it was classified as “likely pathogenic” variant.

**FIGURE 2:**
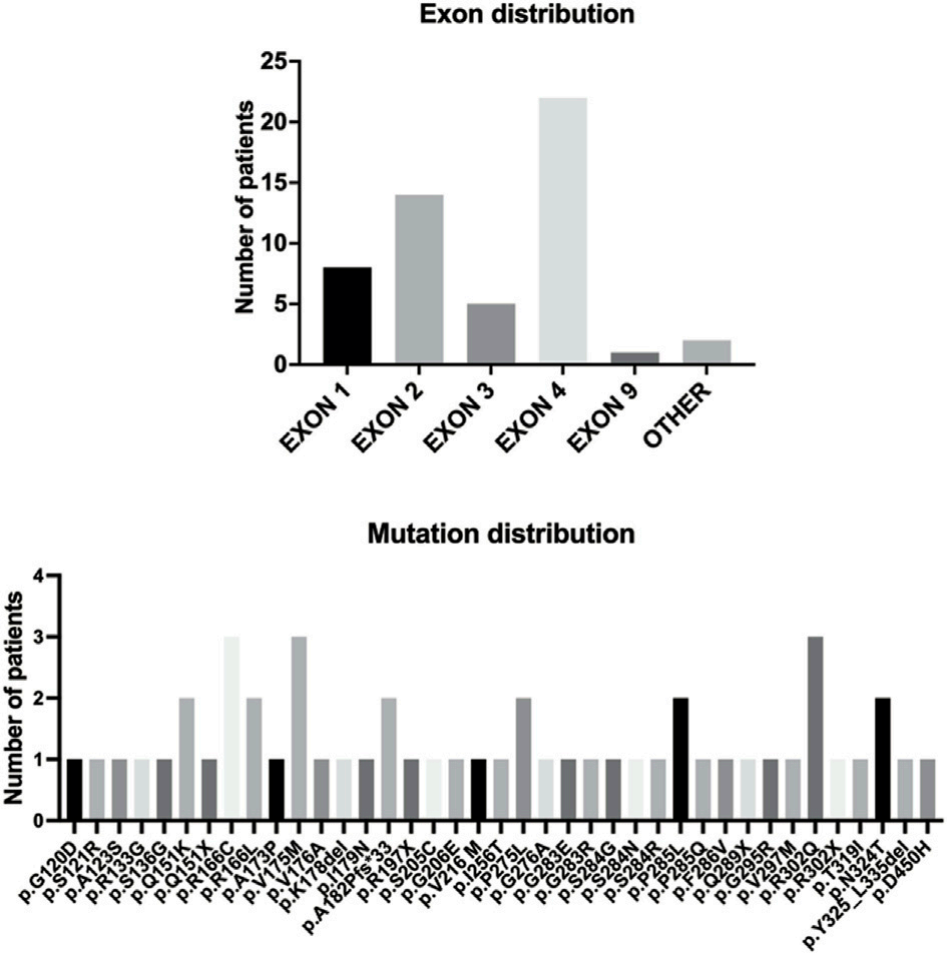
The *HTRA1* mutations identified in patients with *HTRA1*-associated dominant cerebral small vessel disease. The bar chart above represents the exon distribution of identified heterozygous *HTRA1* mutations. The bar chart below represents the identified heterozygous *HTRA1* mutations so far.

The detailed information of the five variants are listed in Table 1. Among them, three were in exon 4, one in exon 2, and the splicing variant was in intron 1. Combined with previous reports, heterozygous *HTRA1* mutations were clustered in exon 1-4 especially in exon 4 (Figure 2). Our work provided further evidence of “hot regions” for heterozygous *HTRA1* mutations, Moreover, HTRA1 variants c.496C>T, c.824C>T, c.854C>T, c.883G>A, c.889G>A, and c.904C>T in both heterozygous and homozygous cases were evidenced.

**TABLE 1:** Heterozygous *HTRA1* variants identified in the familial CSVD probands.

Nucleotide change	Amino acid change	Protein domain	1000G	ExAC	genomAD	MutationTaster	SIFT	Polyphen2	CADD	ACMG
c.472 + 1G>A*	_	_	_	_	_	D	NA	NA	NA	LP (PM1+PM2+PP3+PP5)
c.543delT	p.A182Pfs*33	_	_	_	_	D	NA	NA	NA	LP (PM1+PM2+PM4+PP3+PP5)
c.824C>T	p.P275L	serine protease	_	0.00002	_	D	D	D	D	LP (PM1+PM2+PP3+PP5)
c.854C > T	p.P285L	serine protease	_	_	_	D	D	D	D	LP (PM1+PM2+PP3+PP5)
c.889G>A	p.V297M	serine protease	_	_	_	D	D	D	D	LP (PM1+PM2+PP3+PP5)

*Novel mutation.

NA, not available; 1000G, 1000 genomes project; ExAC, Exome Aggregation Consortium; genomAD, Genome Aggregation Database; CADD, indicates combined annotation dependent depletion; ACMG, American College of Medical Genetics and Genomics; LP, likely pathogenic.

### 3.2 Clinical features

#### 3.2.1 General information of patients

A total of 5 probands carrying heterozygous *HTRA1* pathogenic variants were detected including 4 males and one female. All cases had a positive family history of ischemic stroke. All probands mimicked typical symptoms of CARASIL except case 5. Among these 5 probands, 4 had cognitive decline with variable onset age ranging from 44 to 61 years old (mean age at onset was 52.5 years). Four probands experienced ischemic stroke and the average age of first ischemic attack was 49 (ranging from 44 to 53 years old). Four probands suffered from alopecia at their thirties or forties. Two probands experienced spondylosis or lumbago at their forties. Gait disturbance was observed in two patients. Mood disorder was found in three probands. None of them had migraine. In family 1, the same *HTRA1* mutation was detected in the son of the proband, who experienced obvious alopecia and an acute lumbago attack at his thirties. The son of proband 2, harboring the same mutation as his father, suffered from an acute lumbago at his twenties and was diagnosed with lumbar disk herniation. The detailed clinical characteristics of patients carrying heterozygous *HTRA1* mutations are summarized in Table 2.

**TABLE 2:** Main clinical characteristics of the heterozygous *HTRA1* pathogenic variant carriers.

Patients	F1 (II-3)	F1 (III-2)	F2 (II-1)	F2 (III-1)	F3 (II-2)	F4 (II-5)	F5 (II-4)
Mutation	c.472 + 1G>A	c.472 + 1G>A	c.824C>T	c.824C>T	c.854C >T	c.543delT	c.889G>A
Sex	M	M	M	M	F	M	M
Age	62	36	53	26	44	57	62
Onset (y)	31	30	33	23	32	40	51
Vascular risk factors	Hypertension	_	_	_	Hypertension	-	Hypertension
Symptoms at onset	Alopecia	Lumbago	Alopecia	Lumbago	Alopecia	Alopecia	Stroke
TIA/stroke	_	_	+	_	+	+	+
Cognitive decline	_	_	+	_	+	+	+
Mood disorder	_	_	_	_	+	+	+
Alopecia	+	+	+	_	+	+	_
Lumbago	+	+	+	+	_	_	_
Gait disturbance	_	_	_	_	+	_	+
Stenosis of IEV	+	+	+	+	+	+	+
White mater lesions	+	NA	+	NA	+	+	+
Multiple lacunar foci	+	NA	+	NA	+	+	+
Microbleeds	_	NA	+	NA	+	+	NA
Affected relatives	3	3	2	2	4	2	4

+Indicates yes.

−Indicates no.

NA, not available; F, female; M, male. IEV, intra-extracranial vessels.

#### 3.2.2 Clinical features of patients carrying heterozygous *HTRA1* mutations

**FIGURE 3:**
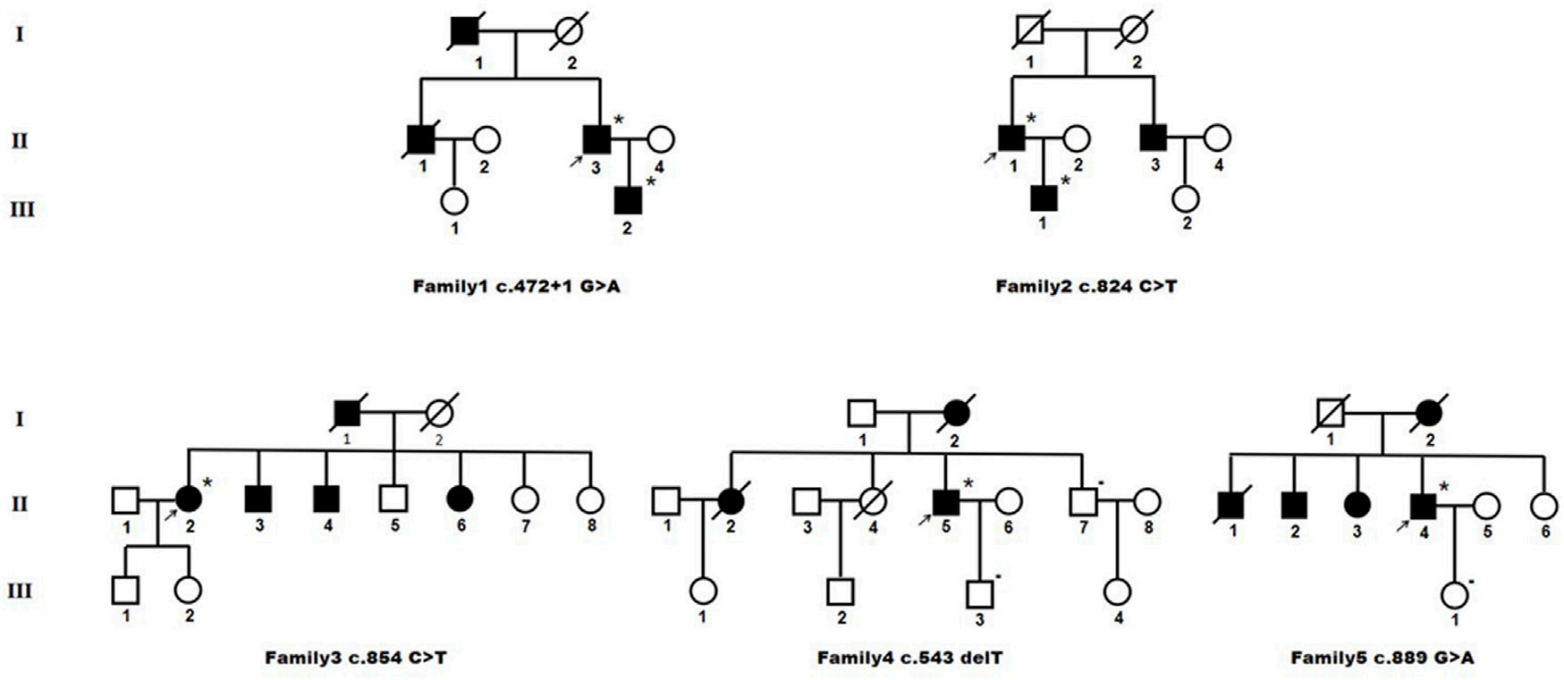
Pedigree charts of heterozygous *HTRA1* pathogenic variant families. Squares indicate males; circles indicate females; the black symbols indicate affected individuals; *indicates patient with single *HTRA1* pathogenic variant; - indicates patient without *HTRA1* pathogenic variant; arrows indicate the probands.

Pedigree charts of the patients with *HTRA1* mutations were seen in Figure 3. Their detailed clinical features were described as following.

##### 3.2.2.1 Family 1

The proband (II-3) with c.472 + 1G>A variant is a 62-year-old man, experienced alopecia in his thirties. He suffered from an acute lumbago at his forties and was diagnosed with lumbar disk herniation. No obvious cognitive decline and mood depression was present. Brain MRI showed multiple lacunars, severe subcortical leukoencephalopathy, and microbleeds. CTA of intra-extracranial vessels showed stenosis of several vessels. The proband had a history of hypertension for 2 years without other vascular risk factors. His dead father (I-1) had a history of cerebral infarction and alopecia. His old brother (II-1) had a medical history of lumbago and was died of ischemic stroke. His 36-year-old son (III-2) experienced obvious alopecia at his thirties and suffered from an acute lumbago several years ago with a diagnosis of lumbar disk herniation.

##### 3.2.2.2 Family 2

The proband (II-1) with c.824C>T (p.P275L) was a 53-year-old man with progressive alopecia since thirties. He had a history of spondylosis and lumbago. Cerebral infarction occurred at age of 53 with cognitive impairment. Brain MRI showed diffuse leukoencephalopathy, multiple lacunars, and microbleeds. The cervical CTA showed mild to moderate stenosis. Brain MRA was normal. There were no vascular risk factors of this patient. His father (I-1) died in the patient’s teens without definite causes. His younger brother (II-3) had a history of cerebral infarction and was not available for genetic test. His 26-year-old son (III-1) had a history of spondylosis and lumbago.

##### 3.2.2.3 Family 3

The index case (II-2) with c.854C>T (p.P285L) had progressive alopecia since thirties. She had her first cerebral infarction at 41. At the age of 44, she had experienced recurrent transient ischemic attack (TIA), mood disturbance, and cognitive impairment. Brain MRI showed leukoencephalopathy with external capsule involvement, multiple lacunars, and several microbleeds. The CTA of cervical arteries was normal. Brain MRA revealed multiple mild stenosis of intracranial vessels. She had a four-year history of hypertension. No diabetes, smoking, hyperlipemia was presented. Her father (I-1) was died of cerebral vascular disease. Her two brothers (II-3 and II-4) and one sister (II-6) had a history of cerebral infarction. Her 26-year-old son (III-1) had an experience of lumbago.

##### 3.2.2.4 Family 4

The proband (II-5), a 57-year-old man, had his first ischemic stroke with dizziness 10 years ago. He carried c.543delT (p.A182Pfs*33) mutation. A second ischemic stroke attacked when he was 57, accompanied by dizziness, nausea and vomiting, dysarthria, and dysphagia. DWI images showed acute lacunar ischemic lesions. Cognitive decline and psychiatric disorders were noted since several years ago. He had alopecia from his forties with a history of cervical spondylosis. Brain MRI showed severe diffuse leukoencephalopathy with external capsule and temporal pole involvement, multiple lacunars and diffuse microbleeds. The CTA of cervical arteries showed mild stenosis. Brain MRA revealed multiple stenosis of intracranial vessels. There were no vascular risk factors of this patient. His mother (I-2) had a history of dementia and was died of cerebral infarction at the age of 66. His older sister (II-2) died of ischemic stroke. Another older sister (II-4) died of lung cancer at her forties without the history of stroke or cognitive impairment. His younger brother (II-7) was asymptomatic.

##### 3.2.2.5 Family 5

The proband (II-4) was a 62-year-old man who suffered from recurrent cerebral infarction for 3 years and progressive memory deficits for 1 year. He had light cigarette smoking history and one-year history of hypertension. During the past 3 years, he suffered cerebral infarction for six times although he took oral antiplatelet drugs regularly. He exhibited emotional lability and vague speech. In addition, he presented progressive memory deficits for 1 year. No headache or lumbar disc herniation was complained. Neurological examinations revealed mild right hemiparesis with brisk tendon reflexes, left facial weakness, slight left limb ataxia, and slurred speech. Alopecia or baldness was not observed. His spine MRI showed physiological cervical and lumbar lordosis, without signs of spondylosis. Brain MRI showed multiple acute cerebral infarction lesion in the left frontal parietal lobe, diffuse and confluent white matter alterations in periventricular area, bilateral frontal lobe, and corpus callosum. In addition, multiple remote lacunar lesions in basal ganglia, brainstem, and periventricular area were seen in the T2-weighted MRI. Temporal lobes were only slightly affected.

However, cervical CTA showed severe stenosis at the origin of left internal carotid artery. Brain CTA showed multiple stenosis in intracranial vessels. After his seventh cerebral infarction, endovascular stent placement was performed for his left ICA. Unfortunately, he developed large area cerebral infarction and cerebral hemorrhage 2 days after the interventional treatment. His mother (I-2) was died of cerebral infarction with a history of dementia. One of his older brothers (II-1) was died of cerebral infarction. The other (II-2) manifested mild cognitive decline. His older sister (II-3) had a medical history of ischemic stroke. Genetic analysis of the proband revealed a heterozygous *HTRA1* c.889G>A (p.V297M) variant.

## 4 Neuroimaging features

### 4.1 Brain MRI findings

**FIGURE 4:**
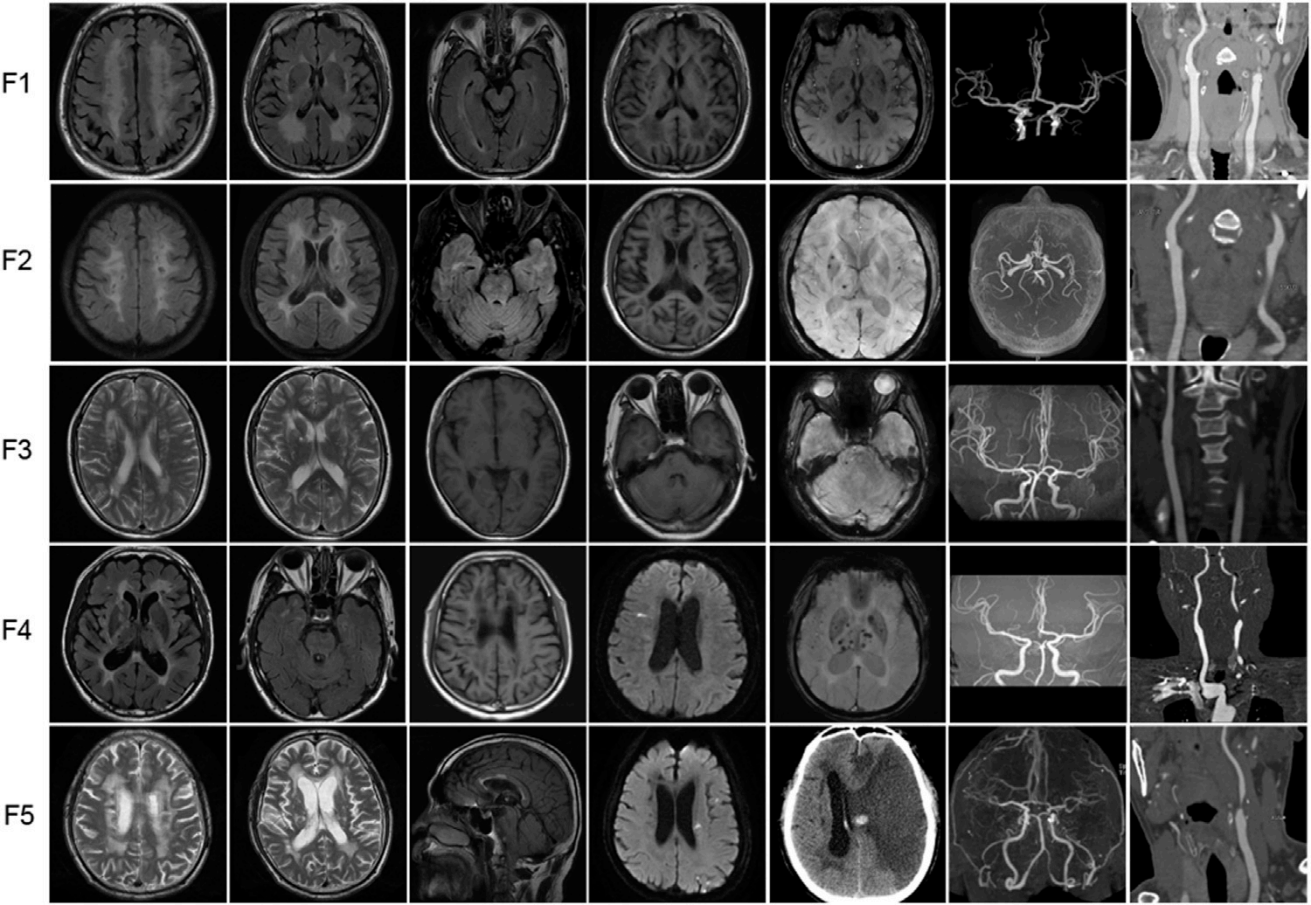
Representative neuroimages of the probands with heterozygous *HTRA1* pathogenic variants. T2-weighted images or fluid attenuation inversion recovery images of brain MRI to illustrate white matter hyperintensity. T1-weighted images or fluid attenuation inversion recovery images of brain MRI to illustrate lacunar infarcts. DWI images showed acute lacunar cerebral infarction. SWI images to illustrate microbleeds. Brain MRA or CTA to illustrate stenosis of intracranial vessels. Cervical CTA to illustrate stenosis of cervical vessels. For F5 (patient II-4), cervical CTA showed severe stenosis at the beginning of left internal carotid artery. Brain CT after interventional treatment showed cerebral hemorrhage and large area cerebral infarction.

The brain MRI of 5 probands with heterozygous *HTRA1* pathogenic variants was demonstrated in Figure 4. All the five patients showed subcortical white matter lesions and multiple lacunar ischemic lesions while 4 patients presented microbleeds. As for white matter lesions, periventricular and frontal area was involved in all the 5 probands while centrum semiovale and thalamus involvement was observed in 4 patients. Four probands presented corpus callosum involvement. Anterior temporal lobes involvement of white matter was found in two patients and external capsule in three. The “arc sign” (the arc-shaped hyperintensity from the pons to the middle cerebellar peduncles), a characteristic finding for CARASIL in the advanced stage, was not observed in any one of them (Nozaki et al., 2015). Their U-fibers were all spared. DWI images showed acute lacunar ischemic lesions in two patients.

### 4.2 MRA or CTA findings

All the 5 probands exhibited stenosis of intracranial or cervical vessels (Figure 4). Four patients showed mild to moderate multiple stenosis of intracranial vessels by brain MRA or CTA (F1, F3, F4, F5). Another 4 probands exhibited mild to severe stenosis of cervical vessels (F1, F2, F4, F5).

## 5 Discussion

Up to this study, there have been 40 single heterozygous *HTRA1* mutations (Figure 2) (Verdura et al., 2015; Ihara, 2016; Nozaki et al., 2016; Bougea et al., 2017; Di Donato et al., 2017; Ito et al., 2018; Kono et al., 2018; Lee et al., 2018; Pati and Battisti, 2018; Thaler et al., 2018; Wu et al., 2018; Zhang et al., 2018; Favaretto et al., 2019; Liu et al., 2020; Ohta et al., 2020; Oluwole et al., 2020; Zhuo et al., 2020; Bekircan-Kurt et al., 2021; Grigaitė et al., 2021; Shang et al., 2021; Cao et al., 2022). In this study, we collected 95 Chinese CSVD patients and performed genetic analysis in the probands. After verification by Sanger sequencing, we detected five heterozygous *HTRA1* mutations in five index cases including four previously reported mutations (c.543delT, c.824C>T, c.854C>T, c.889G>A) (Tikka et al., 2014; Nozaki et al., 2016; Lee et al., 2018; Bekircan-Kurt et al., 2021) and one novel mutation (c.472 + 1G>A). Among them, c.854C>T, c.824C>T, and c.472 + 1G>A were reported in Chinese population for the first time and c.889G>A was first reported in a heterozygous case. Based on the following reasons, we consider these 5 heterozygous *HTRA1* variants are pathogenic. Among them, three variants (c.824C>T, c.854C>T, c.889G>A) are predicted to be deleterious by multiple prediction software. The truncated variant c.543delT results in complete or partial loss of the serine protease domain (Lee et al., 2018). The splicing variant c.472 + 1G>A was predicted to be deleterious by Mutation Taster. Human Splicing Finder reveals that this variant causes the alteration of the WT donor site and most probably affects splicing. Besides, they are absent in 200 in-house controls. At molecular level, these variants are mapped to mutation hotspots of *HTRA1*, which are critical functional domains and evolutionally conserved across species. Previous studies indicated that c.889G>A reduced the level of serine protease activity of *HTRA1*, which led to impaired suppression of TGF-β signaling (Hara et al., 2009). Heterozygous c.854C>T mutation resulted in markedly decreased protease activities and impaired *HTRA1* activation cascade *in vitro* study (Nozaki et al., 2016). *HTRA1* encodes a serine protease, and its functional unit is a trimer which is stabilized by residues of the protease domain (Clausen et al., 2002). Mutation c.543delT leads to premature stop codons and *HTRA1* truncation, leading to complete or partial loss of the serine protease domain (Lee et al., 2018). Thus, variant c.543delT may lead to a haploinsufficiency. Variant c.543delT may additionally exert a dominant-negative effect on wild-type *HTRA1* protease activity (Lee et al., 2018). In 2016, Roeben et al. illustrated a pathogenic *HTRA1* variant c.1005 + 1G>T in a CARASIL patient (Roeben et al., 2016). Recently, Sun *et al.* illustrated a mutation c.472 + 2T>C in *HTRA1* in a CARASIL patient (Sun et al., 2022). These cases indicate that our splicing variant c.472 + 1G>A could also be pathogenic. From clinical views, these variants were consistent with the clinical characteristics and brain MRI of the patients. Additionally, for family 1 and 2, their sons who carry the heterozygous *HTRA1* variants also develop symptoms similar to the index cases. But we do have to point out that familial co-segregation was not enough in the pedigrees because other familial members refused genetic investigations. All in all, these 5 heterozygous *HTRA1* variants can be classified as “likely pathogenic” according to the guideline of ACMG (Richards et al., 2015). Thus, our results expand the mutation spectrum of *HTRA1* gene. However, one of the limitations of our study is the lack of functional studies for the mutant protein due to technical and material limitations.

Pathologic features of CARASIL are fibrosis of the adventitia and loss of SMC, multilayering and splitting of the elastic lamina, intimal thickening in cerebral small vessels (Ito et al., 2018). Another limitation of our study is the absence of histopathologic studies. To date, only three pathologic examinations have been done and published in patients with heterozygous *HTRA1* mutations, p. G283E, p. R302Q and p. Q151X (Nozaki et al., 2016; Ito et al., 2018; Thaler et al., 2018). The vessels showed similar histological characteristics to those of CARASIL but apparently less severe. There was no granular osmiophilic material (GOM) accumulation at vascular smooth muscle cells (VSMC), which is a typical sign in CADASIL patients. This indicate that patients with heterozygous *HTRA1* pathogenic variants may share the similar mechanisms to those of CARASIL instead of CADASIL.

In our present study, four probands had very similar clinical characteristics to CARASIL. Four probands developed cognitive decline with an average onset age of 52.5. The average age of first ischemic attack was 49 in four probands. This further supported that a later onset age of stroke and cognitive decline (mainly at the fifth to sixth decade of life) were the main features of *HTRA1*-associated dominant CSVD. As for extra-neurological symptoms, early onset of alopecia was detected in four probands and spondylosis or lumbago was detected in two patients. Verdura et al. illustrated that there was no typical extra-neurological symptoms in *HTRA1*-associated dominant CSVD (Verdura et al., 2015). Nozaki et al. illustrated in their study that spondylosis deformans was detected in all their 8 cases and alopecia was detected in 3 (Nozaki et al., 2016). Combined with previous reports, extra-neurological symptoms may be varied. Overall, heterozygous *HTRA1* pathogenic variant carriers showed milder symptoms than that of homozygous *HTRA1* pathogenic variants.

Noticeably, there was stenosis in intra-extracranial vessels in our heterozygous *HTRA1* pathogenic variant patients. CADASIL accompanied with stenosis of intra-extracranial vessels has been reported in several instances (Choi et al., 2005; Kang and Kim, 2015; Zhang and Zhang, 2019). And it was illustrated that, although so far neglected, infarction associated with intracranial arterial stenosis may be one of the clinical manifestations of CADASIL, at least in East Asia (Kang and Kim, 2015). However, stenosis of intra-extracranial vessels has rarely been reported in heterozygous *HTRA1* mutation patients or CARASIL. Our study found stenosis of intracranial or cervical vessels in all the five probands. Four patients showed multiple stenosis of intracranial vessels from mild to moderate levels by brain MRA or CTA. Another four patients showed mild to severe stenosis of cervical vessels by cervical CTA. We cannot rule out the possibility that stenosis was caused by hypertension since three probands had it. However, apart from vascular stenosis, these probands had other symptoms including cognitive impairment, stroke, low back pain, and hair loss, which could not be explained by hypertension. So we speculated that vascular stenosis may also be caused by *HTRA1* heterozygous mutations. Whether *HTRA1* heterozygous mutations correlate with this unusual clinical manifestation needs further investigation.

Brain MRI characteristics of CADASIL patients are subcortical lacunar infarcts, microbleeds, and white matter hyperintensities in external capsule and temporal poles on T2-weighted imaging (Tikka et al., 2014; Wang, 2018). Typical brain MRI characteristic of CARASIL is similar to that of CADASIL (Tikka et al., 2014; Nozaki et al., 2015). At the early stage, white matter lesions are mainly observed in the frontal lobes and external capsule (Nozaki et al., 2015). Anterior temporal lobe involvement could also be observed. The “arc sign,” a hyperintense arc-shaped lesion from the pons to the middle cerebellar peduncles, is a characteristic sign at the advanced stage for CARASIL (Nozaki et al., 2015). However, the U-fibers are usually relatively spared (Tikka et al., 2014). In our heterozygous *HTRA1* mutation carriers, all the five probands showed subcortical white matter lesions and multiple lacunars while four microbleeds. As for subcortical white matter lesions, external capsule involvement was found in three patients. The “arc sign” was not observed. The U-fibers were all spared which is consistent with previous reports (Verdura et al., 2015; Nozaki et al., 2016; Di Donato et al., 2017). Noticeably, anterior temporal lobes involvement of white matter was found in two patients (F2 and F4). Combined with previous reports, white matter lesions in the anterior temporal lobe of heterozygous *HTRA1* mutation carriers could be absent (Verdura et al., 2015; Di Donato et al., 2017) or present (Nozaki et al., 2016; Lee et al., 2018), which cannot be used to distinguish *HTRA1*-associated dominant CSVD from CARASIL or CADASIL. However, corpus callosum involvement which was generally absent in CARASIL and rarely observed in CADASIL was observed in four patients in our study (Di Donato et al., 2017). Consequently, brain MRI features in heterozygous *HTRA1* mutation carriers share common radiological features with CADASIL and CARASIL.

In conclusion, our study expanded the mutation spectrum of *HTRA1* especially in Chinese populations and provided further evidence for “hot regions” in heterozygous *HTRA1* pathogenic variants*.* Our work further supported that patients with heterozygous *HTRA1* pathogenic variants presented with similar but less severe features than CARASIL but in autosomal dominantly inherited pattern. However, there are still a lot to be investigated. The relationship between *HTRA1* heterozygous mutations and intra-extracranial vessel stenosis is not clear. The genotype-phenotype correlation between the area of white matter lesions and the mutation sites remains unknown. The pathogenic mechanisms of heterozygous *HTRA1* mutations need further investigations.

## Data Availability

The datasets for this article are not publicly available due to concerns regarding participant/patient anonymity. Requests to access the datasets should be directed to the corresponding author.
