# Supplementary figures and images for: Identified novel heterozygous HTRA1 pathogenic variants in Chinese patients with HTRA1-associated dominant cerebral small vessel disease
Source: Front Genet. 2022 Aug 10;13:909131. doi: 10.3389/fgene.2022.909131 (PMC9399615; doi:10.3389/fgene.2022.909131)

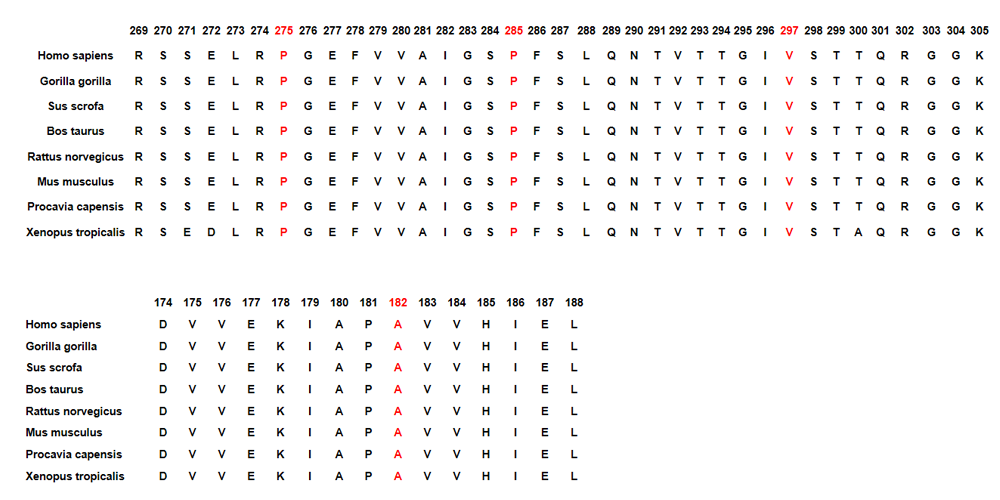

Supplement: Supplementary file 1 [file Image1.TIF]
