# Supplementary material for: Identified novel heterozygous HTRA1 pathogenic variants in Chinese patients with HTRA1-associated dominant cerebral small vessel disease
Source: Front Genet. 2022 Aug 10;13:909131. doi: 10.3389/fgene.2022.909131 (PMC9399615; doi:10.3389/fgene.2022.909131)
Supplement: Supplementary file 2 [file Table3.DOCX]

**Table 3.** The characteristics of the populations in controls and probands

|  | **controls** | **probands** |
| --- | --- | --- |
| Sex |  |  |
| M, n (%) | 112(0.56) | 4(0.80) |
| F, n (%) | 88(0.44) | 1(0.20) |
| Age, yrs, mean | 54.30 | 55.60 |
| Vascular risk factors^#^, n (%) | 24(0.12) | 3(0.60) |
| TIA/stroke, n (%) | 0(0.00) | 4(0.80) |
| Cognitive decline, n (%) | 0(0.00) | 4(0.80) |
| Mood disorder, n (%) | 0(0.00) | 3(0.60) |
| Alopecia, n (%) | 0(0.00) | 4(0.80) |
| Lumbago, n (%) | 2(0.01) | 2(0.40) |
| Gait disturbance, n (%) | 0(0.00) | 2(0.40) |
| Stenosis of IEV, n (%) | 6(0.03) | 5(1.00) |
| White mater lesions, n (%) | 0(0.00) | 5(1.00) |
| Multiple lacunar foci, n (%) | 2(0.01) | 5(1.00) |
| Microbleeds, n (%) | 0(0.00) | 3(0.60) |
| Family history, n (%) | 0(0.00) | 5(1.00) |

F, female; M, male; #, hypertension/ hyperglycemia/ hyperlipidemia; IEV, intra-extracranial vessels; yrs, years.
